# Development and Application of Supported Ionic Liquid Membranes in Microbial Fuel Cell Technology: A Concise Overview

**DOI:** 10.3390/membranes10010016

**Published:** 2020-01-18

**Authors:** Péter Bakonyi, László Koók, Tamás Rózsenberszki, Gábor Tóth, Katalin Bélafi-Bakó, Nándor Nemestóthy

**Affiliations:** Research Institute on Bioengineering, Membrane Technology and Energetics, University of Pannonia, Egyetem ut 10, 8200 Veszprém, Hungary; kook.laszlo4076@gmail.com (L.K.); rozsen88@gmail.com (T.R.); tothgabor2@gmail.com (G.T.); kbbako@gmail.com (K.B.-B.); nemestothy@gmail.com (N.N.)

**Keywords:** ionic liquid, supported ionic liquid membrane, membrane separator, Nafion, microbial fuel cell, bioelectrochemical system

## Abstract

Membrane separators are key elements of microbial fuel cells (MFCs), especially of those constructed in a dual-chamber configuration. Until now, membranes made of Nafion have been applied the most widely to set-up MFCs. However, there is a broader agreement in the literature that Nafion is expensive and in many cases, does not meet the actual (mainly mass transfer-specific) requirements demanded by the process and users. Driven by these issues, there has been notable progress in the development of alternative materials for membrane fabrication, among which those relying on the deployment of ionic liquids are emerging. In this review, the background of and recent advances in ionic liquid-containing separators, particularly supported ionic liquid membranes (SILMs), designed for MFC applications are addressed and evaluated. After an assessment of the basic criteria to be fulfilled by membranes in MFCs, experiences with SILMs will be outlined, along with important aspects of transport processes. Finally, a comparison with the literature is presented to elaborate on how MFCs installed with SILM perform relative to similar systems assembled with other, e.g., Nafion, membranes.

## 1. Introduction

**Figure 1:**
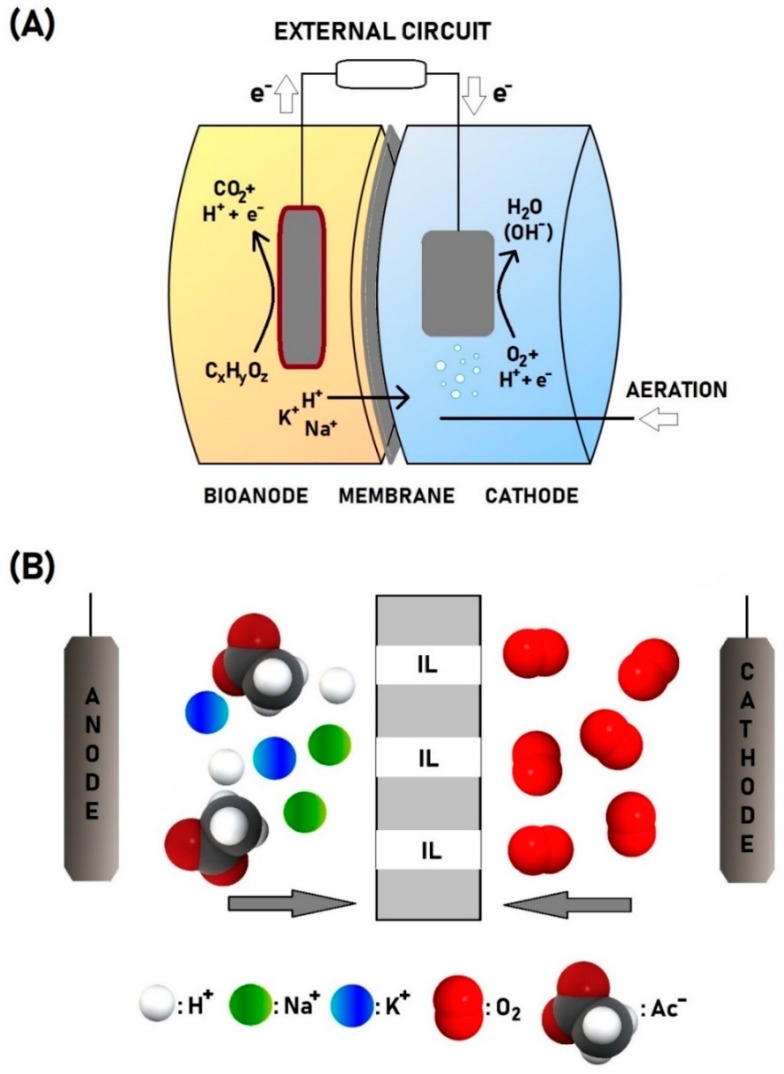
The scheme of a double-chamber microbial fuel cell (MFC) structure and working principles (**A**), and the main components transported through the ionic liquid (IL)-containing membrane (**B**).

Microbial fuel cells (MFCs) represent one particular type of bioelectrochemical system, in which organic matter (e.g., environmental pollutants such as wastewaters) is removed and transformed into electricity [1,2,3]. Architecturally, MFCs (especially those designed in a dual-compartment layout) consist of three substantial elements, namely, the two electrodes (anode and cathode) and a separator—most frequently a membrane—in between them (Figure 1A) [4,5,6]. In fact, their characteristics have an imperative effect on the achievable process efficiency for several reasons, as follows. First of all, electrode properties such as those of the anode affect the development of electrochemically-active biofilms on its surface [7]. Anodes, from the point of view of exoelectrogenic strains, can be seen as terminal electron acceptors utilized under anaerobic conditions [8]. In this sense, anodes are interfaces connecting the biofilm with the (normally abiotic) cathode electrode (as a terminal electron donor, e.g., for oxygen reduction) by receiving the electrons (liberated from microbial metabolic pathways of organic matter oxidation) and conveying the charge to the external circuit containing (a resistor and) the cathode [9,10,11]. Furthermore, to increase the electrochemical efficiency of a bioelectrochemical system such as MFC in terms of the current production and power output, the total internal resistance of the MFC should be reduced [12,13]. This, from the perspective of electrodes, should result in decreased charge transfer resistances and overpotentials [14,15,16].

Besides the electrodes, the traits of the separator (typically a membrane, as mentioned) are of equal importance for the maintenance of ion transfer [6,17,18]. In essence, for an adequate MFC performance, separators need to ensure the selective passage of certain ionic species (mostly protons, instead of competing ions such as Na+ and K+ often found in remarkable quantities in the anolyte, but which is dependent on the type of membrane), while others should be held back (Figure 1B) [19]. This is advantageous for restricting the occurrence of so-called pH-splitting between electrodes, which has been proven to deteriorate the MFC performance [20,21,22]. Furthermore, separators have to prevent the mixing of reactants, in particular, substrates fed to the anode chamber and the oxygen supplied to the cathode (Figure 1B). If these criteria are not fulfilled due to an insufficient mass transfer resistance, the subsequent crossover of substances leads to significant losses and a sub-optimal MFC efficacy [6,23]. On top of that, higher (ionic) conductivity is also favored for the sake of lowered total internal resistance since separators can be considered an Ohmic resistance feature [24,25]. An additional aspect to consider about membrane separators used in MFC is the operating stability. For instance, reliable membrane separators should withstand chemical as well as biological fouling, which presents a threat, especially in the long-term [26,27]. Such impacts may notably alter the initial (physico-chemical) features of the membrane and as a result, cyclic regeneration or even replacement may be inevitable for keeping the MFC in a good condition.

Overall, the above issues have induced intense R&D in the field of separation technology, e.g., membranes to be applied in MFCs, where one of the latest, emerging directions is linked to the employment of ionic liquids (ILs) [28]. ILs are salts, comprised of (inorganic or organic) anion and (organic) cation parts that can be varied to adjust the IL properties, in agreement with the actual demands. Accordingly, ILs are taken into account as tailor-made compounds with broad recognition in various chemical- and biotechnological areas, thanks to their negligible vapor pressure (non-volatility), remarkable ionic conductivity, and wide electrochemical potential window [29].

In the next sections, progress related to the design and use of novel separators prepared with ILs for MFCs in the form of supported ionic liquid membranes (SILMs) will be reviewed to highlight the most crucial findings of this specific subject and enlighten the perspectives of these materials.

## 2. Research Progress with Supported Ionic Liquid Membranes for Microbial Fuel Cell Applications

**Figure 2:**
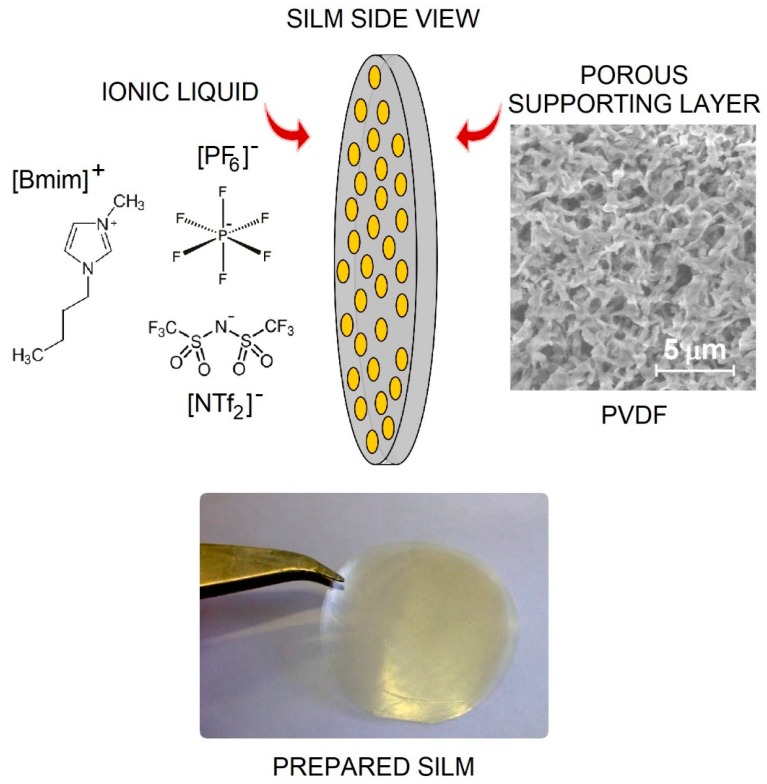
The structure, scheme, and image of a supported ionic liquid membrane (SILM).

Complementing MFCs with supported liquid membrane (SLM) technology has been shown to be effective for increasing the power output of the process [30]. If the SLM is prepared using an ionic liquid, an SILM is eventually obtained. In Table 1, demonstrative examples of using SILM in MFCs are listed. In fact, for membrane fabrication, the ionic liquids comprising imidazolium-type cations have been almost exclusively tested. As for the anion of these ILs, combinations resulting in a lower water miscibility (increased hydrophobicity) are preferred (e.g., [NTf_2_]^−^ and [PF_6_]^−^ instead of [Cl]^−^) in order to act against the extraction of IL from the pores of the support material in an aqueous environment characterizing an MFC. Less variation is noticeable in terms of the porous support material made of either Nylon or PVDF (Figure 2). Since the application of SILM in MFCs is an emerging field, mostly acetate, as a simple, easily degradable substrate, has been used for fundamental studies. The compatibility of the IL with a given support matrix will significantly influence the global stability of the SILM obtained. Nevertheless, besides the purely physico-chemical factors taking place in the half-cells of a dual-chamber MFC, such as mass transport processes and mixing conditions, the interference of SILMs with the underlying microbial culture/consortia (to be seen as the biological component of the MFC) should also be taken into consideration. From this point of view, two main scenarios exist.

On the one hand, if the leakage of ILs from the pores occurs, it will probably affect the activity of electro-active bacteria (EAB). Many ILs have been reported to possess some kind of toxic behavior in various microorganisms. Recently, it was shown by Nemestóthy et al. [31] through a kinetic study that ILs such as [bmim][Cl] and [bmim][Ac], depending on their concentrations, caused a notable loss of metabolic activity of an anaerobic hydrogen-producing community. Furthermore, Hernández-Fernández et al. [32] pointed out that the escape of IL from SILMs to the anolyte contributed to depression of the MFC performance, probably due to perturbation of the whole-cell, electrochemically-active, living biocatalysts. Literature works addressing the stability of SILMs have explained that hydrodynamics in the liquid phase embracing the SILM play a key role and the operation of MFC under static/gently mixed conditions can be seen as a beneficial strategy for enhancing the durability of IL-containing physical separators [33,34,35]. To overcome the instability of SILMs in aqueous media/polar solvents, alternative directions in ionic liquid-containing membrane development for double-compartment microbial fuel cells have appeared. These rely on the blending of various ILs and organic polymers, resulting in a polymer-inclusion membrane, and can be regarded as a possible way forward [36,37,38].

On the other hand, just like in most (filtration) processes where a membrane is employed, chemical and biological fouling of the membrane installed in the bioelectrochemical system is a real threat [26]. Therefore, over time, the deposition of chemical agents, substances, and microbes can be expected. Obviously, the properties of the membrane will then deteriorate, followed by the decrease of the MFC’s power generation capacity. As analyzed in the review of Koók et al. [27] based on previous literature findings, proper selection of the IL to be embedded in the support layer can potentially suppress biofouling. For instance, it has been found by Jebur et al. [39] that liquid membranes prepared with imidazolium cation-based hydrophilic (with [Cl]^−^ and [Br]^−^ anions) and hydrophobic (with [NTf_2_]^−^ anion) ILs considerably lowered the numbers of growing colonies for strains such as *Staphylococcus aureus* and *Pseudomonas aeruginosa*. Therefore, a suitable choice and deployment of ionic liquids could be seen as a potential way to design membranes with a more efficient resistance to microbiological attacks and counteract the biofouling of membrane separators in MFCs [27]. However, due to the limited information available on this aspect, additional research will be needed to assess the traits of biological foulants in light of those of SILMs and understand the possible mechanisms, cross-effects, and interdependencies.

It is worth mentioning that SILMs seem to be advantageous in special bioelectrochemical applications, such as hyper-thermophilic MFCs (>80 °C) [40]. In these systems, extreme thermophilic bacteria serves as biocatalysts; however, common proton exchange membranes (PEM), such as Nafion, fail to stay hydrated for proper functioning. To overcome the issue of the temperature sensitivity of PEMs, Mistry et al. [41] investigated the possible use of ILs in the form of supported liquid membranes. After modifying Nafion and Hyflon PEMs with [bmim][NTf_2_] IL (soaking at different temperatures), SILMs with a high thermal stability and promising anhydrous proton conductivity could be prepared due to successful ion exchange between the proton of –SO_3_H groups of the PEM and the [bmim]^+^ of IL and the relatively high mobility of the H^+^[NTf_2_]^−^ ion pair [41].

## 3. Transport Processes in Ionic Liquid Membranes and at Water/IL Interfaces

As ILs are subject to growing interest for electrochemical applications, including membrane technology, the transport of various compounds into and in the IL phase should be elucidated. In this respect, the transport of (i) water, (ii) IL components, (iii) ionic solutes, and in some cases (iv) gaseous compounds can be addressed.

### 3.1. Mutual Solubility of Water and Hydrophobic Ionic Liquids

An SILM is considered stable when only minimal loss of IL from the porous support to the surrounding aqueous media and a consistent membrane operation are ensured. For this, the first thing to inspect is usually the mutual solubility of water and IL and the influencing factors. In general, ILs with hydrophobic anions are applied for bioelectrochemical applications in the form of a membrane (SILM, in most cases), in order to minimize the water uptake of the liquid membrane and the leakage of IL from the supporting layer. Although the ILs commonly used for such a purpose (prepared by using [PF_6_]^−^, [NTf_2_]^−^, or [DCA]^−^ anions combined, for example, with 1-alkyl-3-methylimidazolium ([C_n_mim]^+^), trioctylmethylammonium ([mtoa]^+^), trihexyl(tetradecyl)phosphonium ([P_666,14_]^+^), and 1-butyl-1-methylpyrrolidinium ([Pyrr_14_]^+^)) are called hydrophobic, they still show more than negligible interactions with water [42,43,44,45]. Moreover, it is known that in addition to the positive effect of a longer alkyl chain length of the cation on the hydrophobicity of IL, mainly the anion defines the hydrophobicity and the extent of miscibility with water [43,44,46,47]. Therefore, the hygroscopic character and sensitivity towards hydrolysis of [PF_6_]^−^ may cause considerable water solubility and simultaneously, the dissolution of IL in the aqueous phase (the latter aspect is minor, but still, the contamination of aqueous phase by IL is best avoided) [34,45]. The [NTf_2_]^−^ anion is also hygroscopic and slightly soluble in water; however, to a much lower extent when compared to [PF_6_]^−^ [48]. It was shown that in SILM prepared with [C_n_mim][PF_6_] (n = 4, 8, 10) being in touch with aqueous phase at both sides, after a given lag-time for reaching the critical water content, continuous water transport occurs [34]. It turned out that water is transferred into the IL phase and then forms clusters or so-called microenvironments, after which a steady permeation of H_2_O can be obtained [34]. Moreover, it can be said that the water present in the IL phase has an effect on the mobility of the cation and anion of ILs, mainly through distraction of the electrostatic interactions between them [49].

### 3.2. Effect of Aqueous Ions on the Mutual Solubility of Water and IL

As the solubility of the salts in aqueous media depends on the presence (and type) of ionic solutes, the effect of the common ions in MFC electrolytes on the IL dissolution to the aqueous phase may be a crucial aspect of SILM stability. MFC anolytes contain a wide spectra of ions from the—mainly wastewater-based—seed source, such as K^+^, Na^+^, Ca^2+^, Mg^2+^, and NH_4_^+^ cations and Cl^−^, SO_4_^2−^, and CH_3_COO^−^ (shortly Ac^−^) anions, as well as ions of the buffering species (HPO_4_^2−^, H_2_PO_4_^−^, HCO_3_^−^, and CO_3_^2−^) or cathodic product (OH^−^) [18].

As was shown for aqueous solutions of water-miscible ILs, the addition of so-called “kosmotropic” (order-making) salts can confine the solubility of ILs in water via the salting-out effect [50,51,52]. To adopt this concept in the field of SILM-based MFCs would be highly beneficial, since the loss of IL from the pores of the supporting layer could be further decreased by moderating the solubility of ILs in water. Freire et al. [53] studied the salting-out of hydrophobic [bmim][NTf_2_] IL from the aqueous phase by using various salts, and concluded that at low salt concentrations (~0.1–0.2 M), a salting-in effect can be observed, followed by salting-out at higher concentrations. Salting-in is manifested in the breaking of the water structure, leading to the stabilization of hydrophobic moieties in the solution by direct ion binding, and thus resulting in an increased solubility of the solute (in our case, IL) in water [53,54]. Since most of the ion concentrations in an MFC range within several mM, it can be said, that under these conditions, SILMs may need to face the salting-in effect—increased solubility in water—in MFCs. However, by using more concentrated buffer solutions (e.g., >200 mM) or applying elevated salt concentrations, the salting-out of IL could be promoted, which could lead to extended SILM stability due to minimized IL loss. However, considering the possible negative effect of a high salt concentration on the biological activity or the rate of membrane/electrode fouling, realistic concentration limits should be maintained.

For instance, Lefebvre et al. [55] investigated the effect of an elevated NaCl concentration on the performance of two-chambered MFCs, and it was found that increasing the amount of NaCl can be tolerated by anodophilic bacteria and boost MFC efficiency up to a given value (~340 mM). Following this point, a further raise in concentration caused significant performance losses. By taking into account the contribution of various ions to the salting-out of IL from water, it can be said that an MFC operation at elevated H_2_PO_4_^−^, PO_4_^3−^, and SO_4_^2−^ concentrations (100–200 mM) may be feasible and advantageous, while higher Na^+^, K^+^, Ca^2+^, and Mg^2+^ concentrations may suppress the solubility of water in the IL phase (although, this latter aspect shows less significance) [53,56]. In addition, it can be said that high amounts of H^+^ and NH_4_^+^ should be avoided, as these compounds may effectively contribute to the increased mutual solubility of IL and water [53,57].

### 3.3. Transport of Ionic Solutes

The transport of ionic solutes is more complex and the exact mechanisms underlying this process have not been fully clarified, so intense research is currently being undertaken. Nevertheless, separate discussion seems to be needed when the transport of ionic species takes place either (i) at the water/IL interface or (ii) in the IL phase. On the one hand, at immiscible water/IL interfaces, it seems that H^+^ transfers into the IL via so-called void-assisted ion-paired proton transfer, which means that proton transport is facilitated by pairing with hydrophobic anions and filling the voids as a capacitive layer in the interfacial IL phase, as it was found for highly hydrophobic IL with a [P_66614_]^+^ cation and [FAP]^−^ (tris(-pentafluoroethyl)trifluorophosphate) anion [43]. On the other hand, alkali metal cations did not follow such facilitated transfer, as their hydrated ionic radii exceeded the estimated size of the voids (being a consequence of the anisotropic nature of ILs) [43]. In the IL bulk phase, although proton transport becomes hindered by the non-polar alkyl groups of the cations, protons have a higher mobility than other diffusing cationic species. This observation highlights the possible use of ILs in MFCs for the enhanced transfer of protons in the presence of other cations, which could contribute to a better performance and pH balance between the anodic- and cathodic-side electrolytes.

The transport of alkali metal ions seems to depend on the presence of water microenvironments in IL, through which they move via diffusion. The molecular diffusion is determined by the size of the transferring ionic species, as well as the viscosity of the IL [34]. For instance, Na^+^ and Cl^−^ are usually characterized by a low solubility in hydrophobic ionic liquids, and they transfer equimolarly. Therefore, it was concluded that small solute transport is not influenced by the selectivity of the IL towards them [34,58]. However, as was found in the case of different forms of thymol blue as a solute, the transfer of larger molecules may be affected by the affinity of IL in relation to them [34]. This mechanism may be of interest for MFCs in terms of anodic organic compounds (as agents interfering with the cathode-surface reactions due to crossover), including substrates. It is also important to note that the presence of various ions in aqueous media may alter the mutual solubility of water and IL [53,57]. In conclusion, it is obvious that ILs provide a special electrolyte media for ion (and related water) transfer, which could be of great interest for MFCs. Therefore, further R&D in this field can be proposed.

## 4. Outlook and Perspectives of SILMs in MFCs

In agreement with the previous discussion, mass transport features across the SILMs ought to be determined for MFC process characterization, especially in terms of (i) the substrate, (ii) ions, and (iii) oxygen, which is the most common reactant at the MFC cathode (Figure 1B). In the case of the oxygen mass transfer properties of membranes, for instance, it was argued that membranes enabling too highly dissolved O_2_ fluxes will eventually make the MFCs work in a sub-optimal way. Suggestions regarding the critical values of oxygen mass transfer coefficients (k*_O_*) have been suggested in papers such as those by Bakonyi et al. [6] and Koók et al. [23]. Inadequate electricity generation by MFC will be directly reflected in measures of electrochemical performance, among which the power density (*P_d_*, mW m^−2^ anode surface) is the most common one.

**Figure 3:**
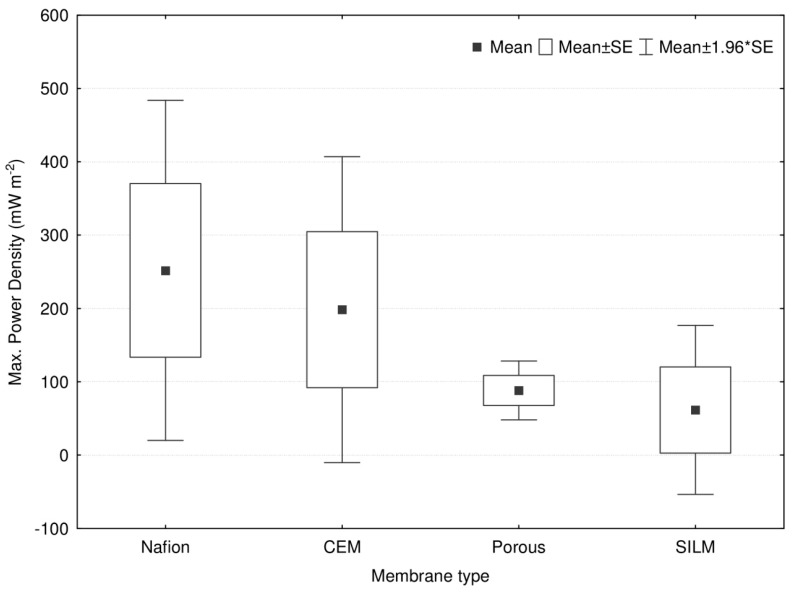
Illustrative comparison of MFCs based on the data of Table 2 with regard to the type of membrane used.

In terms of *P_d_* by MFCs applying SILM as separator, it can be deduced that values fluctuate remarkably. For instance, the maximal *P_d_* derived from total cell polarization tests can vary from 1.4 [25] to 179 mW m^−2^ [33]. It is noteworthy, however, that such improvements may not only be associated with the actual type of IL, e.g., [bmim][PF_6_] instead of [bmim][NTf_2_] on the same PVDF support membrane (Table 1), but are also likely dependent on changing other factors of the MFC architecture, as well as inoculum sources from which EABs are enriched. As the ultimate aim of manufacturing and testing SILMs for MFC is to provide promising candidates as alternatives to the Nafion PEM (proton exchange membrane), a comparative evaluation can show how far the research on and development of SILMs have reached. As a matter of fact, experiences with Nafion membranes in MFCs (acetate substrate, mixed culture as the seed source, dual-chamber configuration, and batch mode) indicate that the maximum *P_d_* could be in a similar range—14.4 [59], 17.7 [60], 38 [61,62], 43.6 [63], 57.5 [64], 65 [65], 118 [66], 126.7 [67], and 173.3 [68]—to that attainable in MFCs with SILMs (1.4–179 mW m^−2^) [23,25,33]. To extend the comparative evaluation of SILMs, it is worth taking a look at the performances of MFCs operated with membrane candidates (such as cation exchange membranes (CEM) and porous (cheap) materials) proposed to compete with Nafion. In Table 2, such a compilation of the literature can be observed for systems with similar underlying working principles. The studies listed in Table 2 have been screened and selected after a careful search of the literature, following certain guidelines and filters suggested by Ge et al. [69] and Whitaker et al. [70]. The main steps were (i) the choice of database: SCOPUS; (ii) the determination of keywords: e.g., “bioelectrochemical system”, “microbial fuel cell”, “membrane”, and “separator”; and (iii) a manual check of the relevance and availability of essential data to be assessed (configuration, inoculum, substrate, operating mode, and maximum power density). In Figure 3, no real differences are detectable and thus, it seems that all MFCs, regardless of the type of membrane separator, could produce comparable maximum power densities. This conclusion is supported by the outcomes of the Tukey hones significant difference (HSD) test presented in Table 3, where no values of *p* < 0.05 (criteria of significant difference) could be noted for any pairs of membrane categories. However, these statistical results should be treated with care due to the lower number of data and should be revisited in the future on a bigger population of samples.

Accordingly, SILMs can be taken into account as plausible separators for microbial fuel cells. However, due to the early-stage of research on these materials (covering approximately a period of 4–5 years), further feedback will be advantageous to reveal their pros and cons during application, especially those related to their stability, biofouling resistance, and simultaneous contribution to MFC efficiency.

## 5. Conclusions

In this paper, the progress that has been achieved with regards to microbial fuel cells operated using membranes containing ionic liquids has been overviewed. It has been shown that the mass transport processes taking place across a membrane and how supported ionic liquid membranes may contribute to efficiently running the process have to be considered in MFCs. Ionic liquids and support materials for the fabrication of SILMs were evaluated in light of literature experiences and a comparative assessment with other membrane-assisted MFCs demonstrated the potential of SILMs as alternative separator candidates for this kind of bioelectrochemical system.

## Figures and Tables

**Table 1:** SILMs used in various MFC studies.

Configuration	Inoculum	Substrate	SILM		Reference
			Ionic Liquid	Support Layer	
Single-Chamber	Mixed Culture	Wastewater	[mtoa][Cl]	Nylon	[32]
			[omim][NTf_2_]	Nylon	
			[omim][BF_4_]	Nylon	
			[omim][PF_6_]	Nylon	
Dual-Chamber	Mixed Culture	Acetate	[hmim][PF_6_]	PVDF	[25]
			[bmim][NTf_2_]	PVDF	
Dual-Chamber	Mixed Culture	Acetate	[hmim][PF_6_]	PVDF	[23]
			[bmim][NTf_2_]	PVDF	
Dual-Chamber	Mixed Culture	Acetate	[bmim][PF_6_]	PVDF	[33]

**Table 2:** Comparative table for MFCs operated with various types of membranes.

Configuration	Operating Mode	Inoculum	Substrate	Membrane Type	Maximum Power Density (mW m^−2^ anode)	Reference
Dual-chamber	batch	Mixed culture	Acetate	Nafion 117	107.9	[71]
Dual-chamber	batch	Mixed culture	Acetate	Nafion 117	38.0	[61]
Dual-chamber	batch	Mixed culture	Acetate	Nafion 117	65.0	[65]
Dual-chamber	batch	Mixed culture	Acetate	Nafion 117	38.0	[62]
Dual-chamber	batch	Mixed culture	Acetate	Nafion 117	1013.0	[72]
Dual-chamber	batch	Mixed culture	Acetate	Nafion 117	118.0	[66]
Dual-chamber	batch	Mixed culture	Acetate	Nafion 117	1225.0	[73]
Dual-chamber	batch	Mixed culture	Acetate	Nafion 117	43.6	[63]
Dual-chamber	batch	Mixed culture	Acetate	Nafion 117	17.7	[60]
Dual-chamber	batch	Mixed culture	Acetate	Nafion 117	126.7	[67]
Dual-chamber	batch	Mixed culture	Acetate	Nafion 117	57.5	[64]
Dual-chamber	batch	Mixed culture	Acetate	Nafion 177	173.3	[68]
Dual-chamber	batch	Mixed culture	Acetate	CEM	33.0	[62]
Dual-chamber	batch	Mixed culture	Acetate	CEM	902.0	[72]
Dual-chamber	batch	Mixed culture	Acetate	CEM	112.0	[66]
Dual-chamber	batch	Mixed culture	Acetate	CEM	114.0	[66]
Dual-chamber	batch	Mixed culture	Acetate	CEM	82.0	[66]
Dual-chamber	batch	Mixed culture	Acetate	CEM	12.6	[60]
Dual-chamber	batch	Mixed culture	Acetate	CEM	320.0	[74]
Dual-chamber	batch	Mixed culture	Acetate	CEM	11.3	[75]
Dual-chamber	batch	Mixed culture	Acetate	Porous	5.0	[62]
Dual-chamber	batch	Mixed culture	Acetate	Porous	36.0	[62]
Dual-chamber	batch	Mixed culture	Acetate	Porous	36.0	[62]
Dual-chamber	batch	Mixed culture	Acetate	Porous	121.0	[66]
Dual-chamber	batch	Mixed culture	Acetate	Porous	114.0	[66]
Dual-chamber	batch	Mixed culture	Acetate	Porous	74.0	[66]
Dual-chamber	batch	Mixed culture	Acetate	Porous	117.0	[66]
Dual-chamber	batch	Mixed culture	Acetate	Porous	41.6	[63]
Dual-chamber	batch	Mixed culture	Acetate	Porous	5.4	[75]
Dual-chamber	batch	Mixed culture	Acetate	Porous	246.7	[67]
Dual-chamber	batch	Mixed culture	Acetate	Porous	163.9	[76]
Dual-chamber	batch	Mixed culture	Acetate	Porous	97.0	[64]
Dual-chamber	batch	Mixed culture	Acetate	SILM	179.0	[33]
Dual-chamber	batch	Mixed culture	Acetate	SILM	4.2	[23]
Dual-chamber	batch	Mixed culture	Acetate	SILM	1.4	[25]

**Table 3:** The *p*-values derived from Tukey’s HSD test for the assessment of significance.

Membrane Type	Nafion	CEM	Porous	SILM
Nafion	-	0.976	0.510	0.735
CEM	0.976	-	0.834	0.895
Porous	0.510	0.834	-	0.998
SILM	0.735	0.895	0.998	-
